# LOSARI: A novel R-based statistical software to facilitate students’ self-regulated learning in statistics courses

**DOI:** 10.1016/j.mex.2025.103739

**Published:** 2025-11-26

**Authors:** Rizal Bakri, Eva Boj del Val, Basri Bado, Ansari Saleh Ahmar

**Affiliations:** a Department of Digital Business, Faculty of Economics and Business, Universitas Negeri Makassar, Makassar, 90224, Indonesia; b Department of Economic, Financial and Actuarial Mathematics, Faculty of Economics and Business, Universität de Barcelona, Barcelona, 08034, Spain; c Department of Development Economics, Faculty of Economics and Business, Universitas Negeri Makassar, Makassar, 90224, Indonesia; d Department of Statistics, Faculty of Mathematics and Natural Science, Universitas Negeri Makassar, Makassar, 90224, Indonesia

**Keywords:** bs4Dash, R programming, Shiny, Self-regulated learning, Statistics Courses

## Abstract

This article presents the development of LOSARI, a novel R-based statistical software designed to facilitate students’ self-regulated learning (SRL) in statistics courses. LOSARI can be accessed online without installation and allows students to perform statistical analyses through a point-and-click interface without coding. It integrates several innovative features: interactive video tutorials embedded in the analysis environment, real-time error notifications that guide students in correcting mistakes, and automatic interpretation of results to support independent learning. The software was validated through a student satisfaction survey using the End-User Computing Satisfaction (EUCS) model, which indicated that most users had positive perceptions of LOSARI and found it effective for learning statistics outside the classroom. Possible extensions and enhancements are also discussed.

• A structured process for developing LOSARI as an R-based statistical learning tool.
• Introduction of key features, including interactive video tutorials, real-time error notifications, and automatic interpretation.
• Validation method through student satisfaction measurement and comparison with manual statistical coding.

## Graphical abstract

**Figure:**
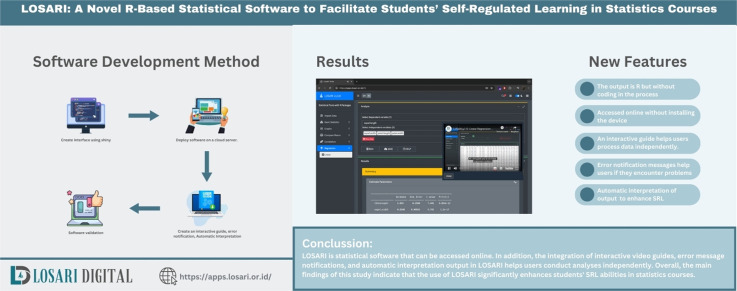

Specifications table

**Table:** 

**Subject area**	Mathematics and Statistics
**More specific subject area**	Statistical tools for learning
**Name of your method**	Losari Digital
**Name and reference of original method**	NA
**Resource availability**	NA

## Background

The concept of self-regulated learning (SRL) has gained increasing significance in modern education, particularly in higher education institutions (HEIs), as it emphasizes students' active participation in organizing, monitoring, and controlling their own learning processes. SRL encompasses techniques, strategies, and metacognitive skills such as self-evaluation, goal setting, strategic planning, self-monitoring, and seeking assistance [[1], [2], [3]]. According to reference [4], SRL's most notable characteristic is the autonomy it grants students, enabling them to manage their learning processes and academic objectives effectively [[5], [6], [7]]. Research indicates that high-performing students tend to exhibit strong SRL behaviors, frequently employ SRL strategies, and demonstrate well-developed self-regulation skills, whereas low-performing students often struggle with SRL and rarely implement its strategies [[8], [9], [10], [11]].

Despite its potential benefits, implementing SRL presents several challenges influenced by contextual and personal factors, including metacognitive, cognitive, and behavioral processes, as well as motivation [5,12]. Additionally, the success of SRL depends on learning strategies, technological support, and course structures. Not all courses effectively integrate SRL outside the classroom. According to reference [13], courses combining theoretical and practical components present unique challenges, as students often require additional guidance [14,15].

Statistical courses, which merge theory with data analysis, pose distinct challenges for SRL. Students often encounter difficulties in interpreting complex statistical concepts and navigating data analysis software. As a result, instructors must repeatedly explain analytical steps in class, yet time constraints limit extensive explanations. Additionally, variations in students' cognitive abilities complicate the learning process, making it difficult to ensure consistent comprehension across the student group. To address these challenges, integrating digital learning tools such as interactive visualization platforms and online practice environments can enhance SRL effectiveness [16,17].

Various statistical software programs, including SPSS, JASP, STATA, SAS, SmartPLS, LISREL, EVIEWS, JAMOVI, Python, and R, are widely used in HEIs for data analysis. However, these tools primarily focus on performing statistical computations and visualization. They rarely provide features that directly facilitate SRL, such as integrated instructional support, contextual guidance, or automated interpretation of results. For instance, coding-based tools like R and Python require programming proficiency, which is not accessible to students from diverse academic backgrounds [17]. R-based point-and-click tools such as JAMOVI, JASP, SWANSTAT, and other Shiny-based applications reduce technical barriers but still separate instructional resources from the software environment, forcing students to switch between external tutorials and the application itself. Although these platforms provide user-friendly interfaces, they focus mainly on statistical computations and visualizations without integrating learning-support features such as interactive tutorials, contextual error handling, or automatic interpretation. To clarify these distinctions, Table 1 presents a brief comparison between LOSARI and existing R-based statistical software, highlighting the features that differentiate LOSARI from other tools.

**Table 1:** Comparison of LOSARI and existing R-based statistical software.

Software	Requires Coding	Integrated Video Guide	Real-Time Error Notification	Automatic Interpretation
JASP (v0.95.3)	No	No	Yes (limited)	No
JAMOVI (v2.7.6)	No	No	Yes (limited)	No
SWANSTAT (v1.0.0)	No	No	No	No
LOSARI (v2.0.0)	No	Yes	Yes	Yes

**Note:** The comparison reflects the current versions of each software at the time of writing. Future updates may introduce similar or extended features.

As shown in Table 1, LOSARI introduces a unique combination of interactive video tutorials, real-time error notifications, and automatic interpretation, features that are not jointly available in existing R-based statistical tools. This distinction highlights the novelty of LOSARI, which was explicitly designed to combine these features within a single environment to better support SRL. According to reference [18], many students avoid using technology for SRL because existing tools lack comprehensive support. However, integrating SRL with advanced educational technologies can have a significant positive impact [19].

To address this gap, this study introduces LOSARI, an educational technology developed using R with the Shiny [20] and bs4Dash [21] packages. LOSARI is accessible online without installation and provides a user-friendly, point-and-click interface that eliminates the need for coding. Its key innovations include the integration of features not available together in existing software: an interactive video tutorial embedded directly within the data processing environment, real-time error notifications that guide students in applying statistical methods correctly, and automated interpretation of statistical results that reduce cognitive load and enhance self-regulated learning. The effectiveness of LOSARI was evaluated using the End-User Computing Satisfaction (EUCS) model, which is widely applied in educational technology research.

## Method details

### Software development method

**Step 1:** Create an interface with the shiny package and deploy on a cloud server.

The first step was to develop R codes for each statistical method and integrate them into a user-friendly web interface using the Shiny framework. In this architecture, Shiny bridges R and the web environment, allowing R scripts to be executed dynamically while displaying interactive results through a browser. The system consists of two core components: the user interface (UI) and the server logic. The UI defines the layout, widgets, and visual elements that users interact with, while the server logic contains the R scripts that perform statistical computations, data processing, and rendering of results. Both components communicate reactively through Shiny’s input–output bindings, ensuring that any change in user input automatically updates the output without reloading the page. This architecture enables LOSARI to deliver immediate visual feedback and perform analyses interactively within a browser-based environment. This design choice is supported by [22], which shows that most users prefer software with a point-and-click interface compared to coding-based tools.

The graphical interface of LOSARI was built using the bs4Dash package [21], an LTE admin dashboard framework based on Bootstrap 4, which was further customized to meet the requirements of this study. For data handling, LOSARI integrates the readr package [33] to upload datasets and the rhandsontable package [34] to display them in an interactive table format. These integrations allow users to import, edit, and view data seamlessly within the application environment, eliminating the need for manual coding. In addition, various other packages are integrated, as shown in Table C.1 in Appendix C.

**Fig. 1:**
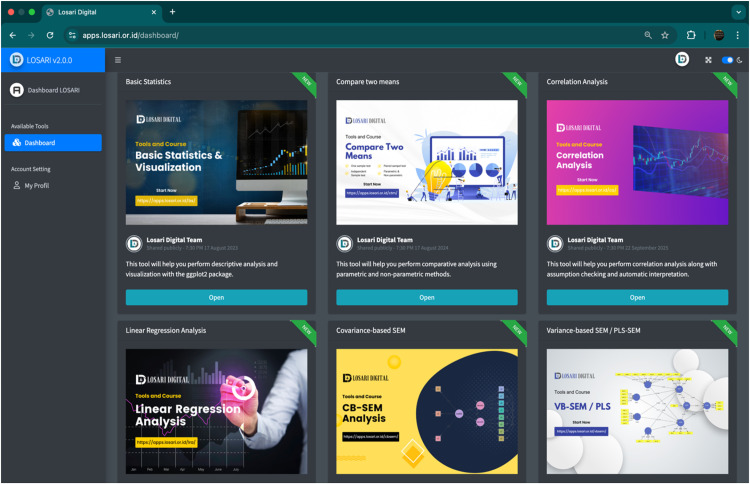
The LOSARI dashboard after successful login, showing the main menu of statistical analysis methods, including Basic statistics, comparison of means, correlation, regression, and additional methods that are under ongoing development. When a user selects one of these methods, a pop-up video guide is automatically displayed to assist in navigating the application.

After implementing the statistical methods and integrating the main learning-support features, including interactive video guides, real-time error notifications, and automated interpretations, the application was deployed to a cloud server using the shiny-server web service. The purpose of this deployment was to ensure that users are not required to install R or Shiny packages locally, thereby making LOSARI more accessible and practical through various updated browsers. LOSARI can be accessed using the latest versions of browsers on both desktop and mobile devices via https://apps.losari.or.id/. Online learning platforms have been shown to provide an effective environment for students to engage in independent learning [23]. Fig. 1 illustrates the LOSARI dashboard after successful login, where various statistical analysis methods are displayed.

**Step 2:** Create an interactive guide, error notification, and automatic interpretation

The second step was to develop interactive guides, real-time error notifications, and automatic interpretation features, which represent the key innovations of LOSARI and distinguish it from other statistical software.

**Fig. 2:**
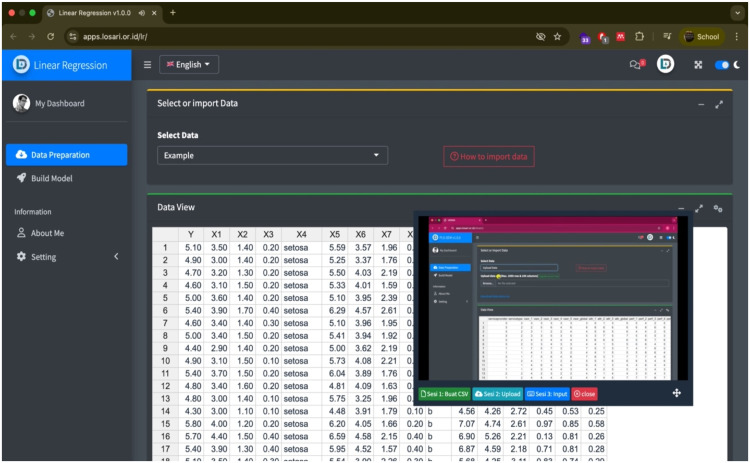
Interactive video tutorial embedded within LOSARI. The tutorial provides step-by-step instructions in a movable pop-up window that remains visible during data processing, enabling students to follow the guide while conducting analyses in the same environment.

The interactive guide video was constructed by recording sequential procedures using LOSARI. The recorded video was uploaded to YouTube, where subtitles were added to improve accessibility. The final version was embedded into LOSARI as a movable pop-up window that remains visible even while users continue data processing in the background. This design enables students to learn by following step-by-step guidance without leaving the analysis environment. Prior studies confirm the effectiveness of video-based learning for self-regulated learning: video media facilitates independent problem-solving [25], enhances student control in digital environments [26], and supports clearer delivery of educational content compared to traditional methods [27]. Specifically in statistics, learning with video tutorials has been shown to improve student performance [28]. Moreover, integrating multimedia in a single multi-screen display helps enhance cognitive processes and learning efficiency [29]. Fig. 2 illustrates an interactive tutorial video embedded within the LOSARI interface.

In addition, LOSARI implements a comprehensive real-time error handling and diagnostic feedback system to enhance both analytical accuracy and users’ conceptual understanding. Error detection is primarily managed on the server side using the *validate(), req()*, and *tryCatch()* functions in R, which continuously monitor user inputs and analytical operations. When an invalid condition is detected, such as incompatible variable types, missing values, or non-numeric inputs for numeric analyses, the system triggers a contextual “toast” notification at the bottom-right corner of the screen. Each message is color-coded (red for critical errors) and provides not only the error description but also corrective suggestions (for example, *“Selected variable must be numeric. Please redefine it in the Data Preparation menu.”*). Another example can be found in the Table D.1 in Appendix D.

**Fig. 3:**
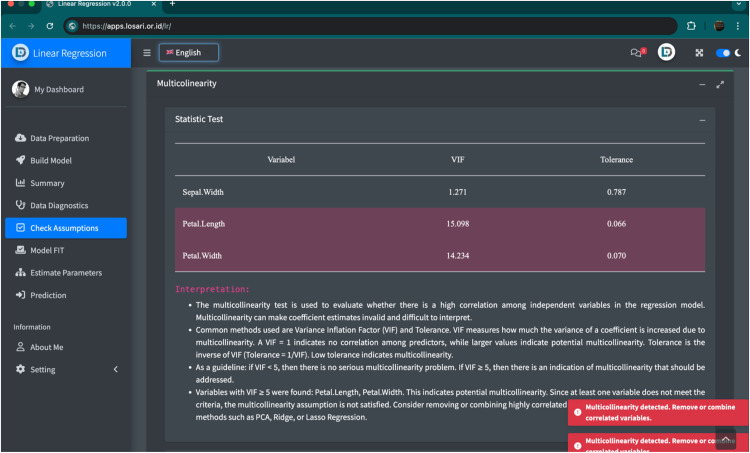
Real-time error notifications are displayed in the lower right corner of the LOSARI interface. This feature alerts users when inappropriate data or variables are selected or when statistics do not meet the criteria and provides feedback by guiding them to correct errors without interrupting the analysis workflow.

Beyond input validation, LOSARI also provides analytical diagnostics that appear dynamically in the output tables. When the system detects statistical issues such as multicollinearity, non-significant test results, or assumption violations, the corresponding cells are automatically highlighted in red, accompanied by brief contextual notes that help users interpret the results accurately. For instance, in regression analysis, variables with non-significant coefficients are visually marked, while a message may indicate that multicollinearity is present. This two-level mechanism ensures that users receive immediate, context-sensitive feedback during both data input and result interpretation. It prevents technical errors and promotes a deeper understanding of statistical reasoning, aligning with the principles of SRL. Prior studies highlight that warnings, dialogs, and tooltips effectively support user focus and task continuity [30]. Fig. 3 presents an example of an error notification message that appears during data analysis. Examples of common error types and corresponding system feedback are provided in Appendix D.

**Fig. 4:**
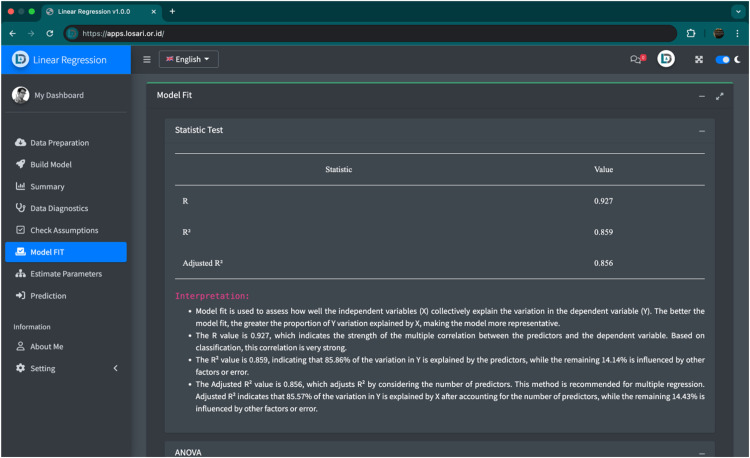
Example of statistical output in LOSARI with automated interpretation. In addition to numerical results presented in tables and plots, the system generates contextual explanations that help students understand the meaning of coefficients, p-values, and other metrics, thereby supporting self-regulated learning.

The final innovation is the automatic interpretation module, which generates textual explanations for statistical outputs such as tables and plots. Instead of presenting only numerical values, LOSARI provides contextual interpretations that explain the meaning of results, thereby reducing cognitive load and supporting higher-order thinking skills. For example, in a regression analysis, LOSARI not only reports coefficients and p-values but also explains whether a predictor has a statistically significant effect and how the direction of the effect should be interpreted. This aligns with [31,32], who argue that practice-based learning combined with in-app guidance enhances self-directed learning and promotes higher-order cognitive skills. Fig. 4 presents an example of statistical output accompanied by automated interpretation.

After developing these core features, the next step was to design a structured user workflow to describe how students interact with LOSARI from data input to result interpretation, as discussed in the following section.

### Software workflow

The workflow of LOSARI follows a structured sequence of user interactions, beginning with access through a various updated browser. Upon visiting the application, users are directed to a login page. After successfully logging in, the dashboard is displayed, providing access to a range of statistical analysis methods, including descriptive statistics, comparison of means, correlation, regression, data preparation, and other techniques available (Fig. 1).

When users select a statistical method for the first time, a pop-up video guide is automatically displayed to assist them in navigating the application. Previous research [24] suggests that providing navigation guidance during the first access significantly improves usability and helps users take the next step more easily. If additional guidance is required before conducting the analysis, users may click the help button to open an interactive video guide. The video appears as a movable pop-up window, enabling students to follow the tutorial without leaving the analysis environment (Fig. 2). This feature supports self-regulated learning by allowing users to access assistance only when necessary.

The next stage involves input, data preparation, and analysis execution. Users can upload their own datasets, view them in tabular form, and apply preprocessing through the gear icon in the top-right corner of the data table. Data preprocessing serves as an initial step to transform raw data into a cleaner and more structured format. Three options are available: define variables, compute variables, and apply filters. The define variables option allows users to modify the data type of variables (e.g., numeric, character, or factor). The compute variables option enables the creation of new variables using arithmetic operations presented through a calculator-style interface, while the filter option allows users to subset rows of data based on specific conditions. Together, these features allow students to prepare data seamlessly within the application without requiring direct R programming.

During or after data preparation, users may proceed with the selected analysis. At this stage, LOSARI provides real-time error notifications to prevent invalid operations, such as selecting incompatible variables for a given statistical method or other input errors that disrupt the process. Notifications appear unobtrusively in the bottom-right corner, guiding users to correct their inputs without interrupting the analysis workflow (Fig. 3).

After successful data processing, LOSARI generates statistical outputs in the form of tables, plots, and interpretation. These outputs are accompanied by an automatic interpretation module, which provides contextual explanations of the results to reduce cognitive load and enhance conceptual understanding (Fig. 4).

**Fig. 5:**
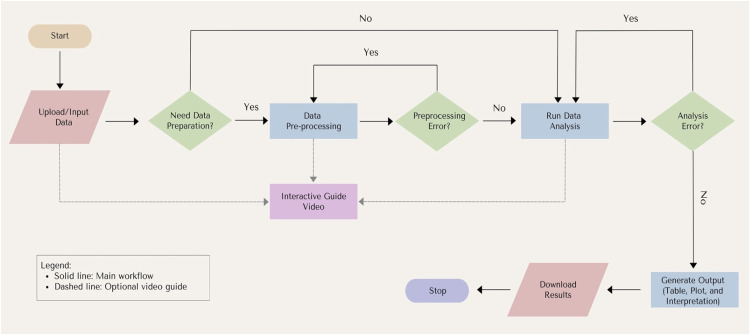
Workflow diagram of LOSARI, illustrating the main workflow from access and login to data preparation, analysis, error handling, output generation, automatic interpretation, and result download. Video guides are shown as optional supporting steps at multiple stages.

Finally, users can download results, including tables, plots, and interpretations, for use in assignments, reports, or further analyses. To summarize, the overall workflow of LOSARI is illustrated in Fig. 5, which highlights the main stages of access, method selection, data preparation, analysis, error handling, output generation, automatic interpretation, and result download. This workflow demonstrates how LOSARI integrates accessibility, contextual guidance, and automated interpretation into a single environment, thereby facilitating students’ self-regulated learning in statistics courses.

## Method validation

### Validation of LOSARI versus R studio outputs

To ensure the reliability of LOSARI, its statistical outputs were compared with those generated by R Studio using the well-known iris *dataset*, which is commonly used in various research studies. Multiple linear regression was selected as the validation case because it involves several key parameters that are commonly reported in statistical modeling. The regression model specified *Sepal.Length* as the dependent variable, *with Sepal.Width, Petal.Length*, and *Petal.Width* as independent variables, formulated as follows:

$$Sepal.Length=\beta_{0}+\beta_{1}\left(Sepal.Width\right)+\beta_{2}\left(Petal.Length\right)+\beta_{3}\left(Petal.Width\right)+\epsilon$$

The corresponding R code used for validation in R Studio is provided below:

data("iris")

fit <- lm(Sepal.Length ∼ Sepal.Width + Petal.Length + Petal.Width, data = iris)

sum <- summary(fit)

sum$coefficients <- round(sum$coefficients, digits = 3)

sum

**Fig. A.1:**
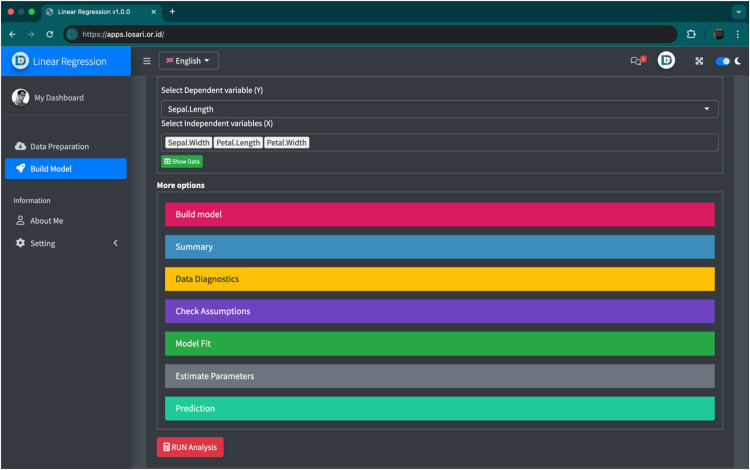
Screenshot of regression setup in LOSARI.

**Fig. A.2:**
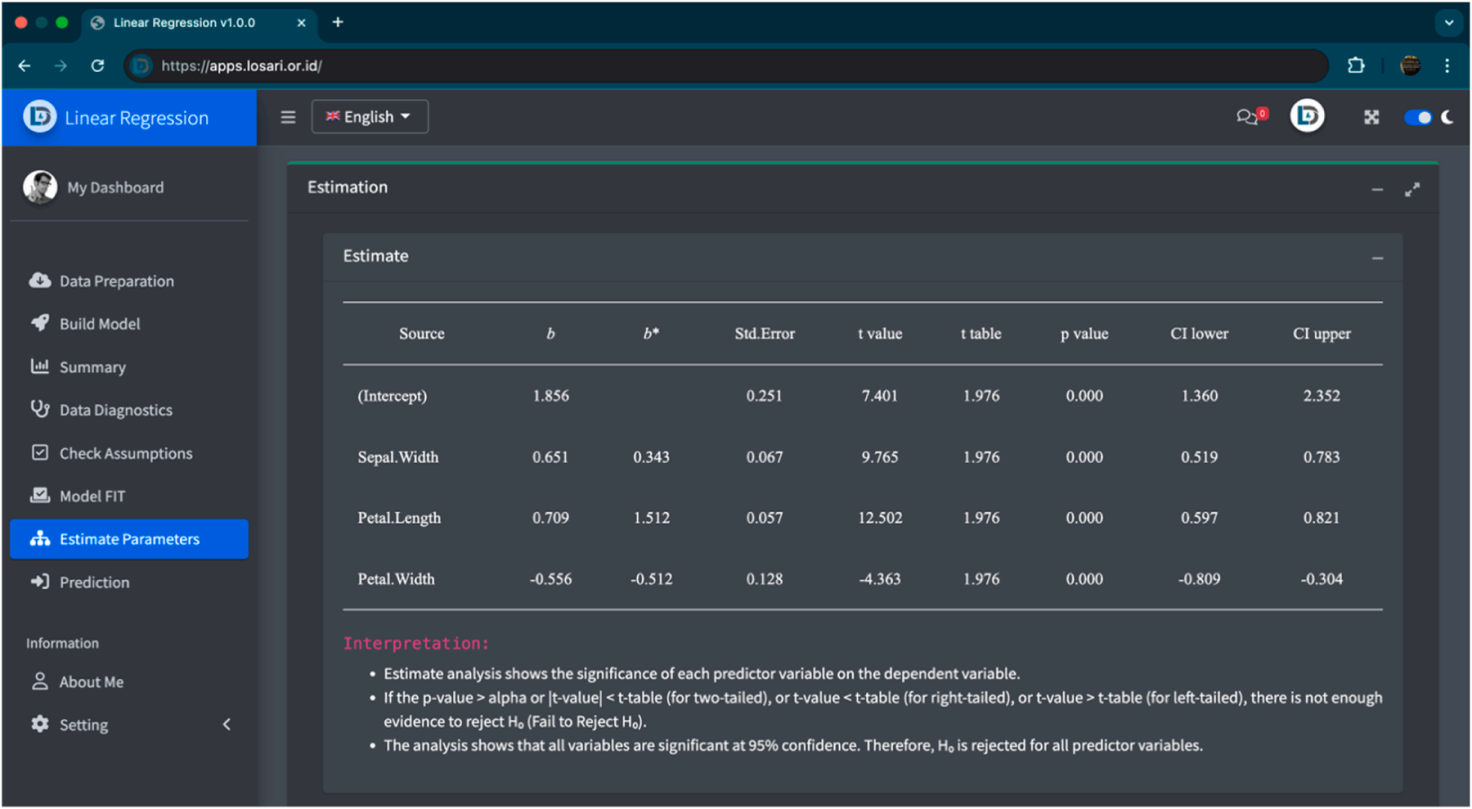
Screenshot of regression output and automatic interpretation in LOSARI.

**Fig. B.1:**
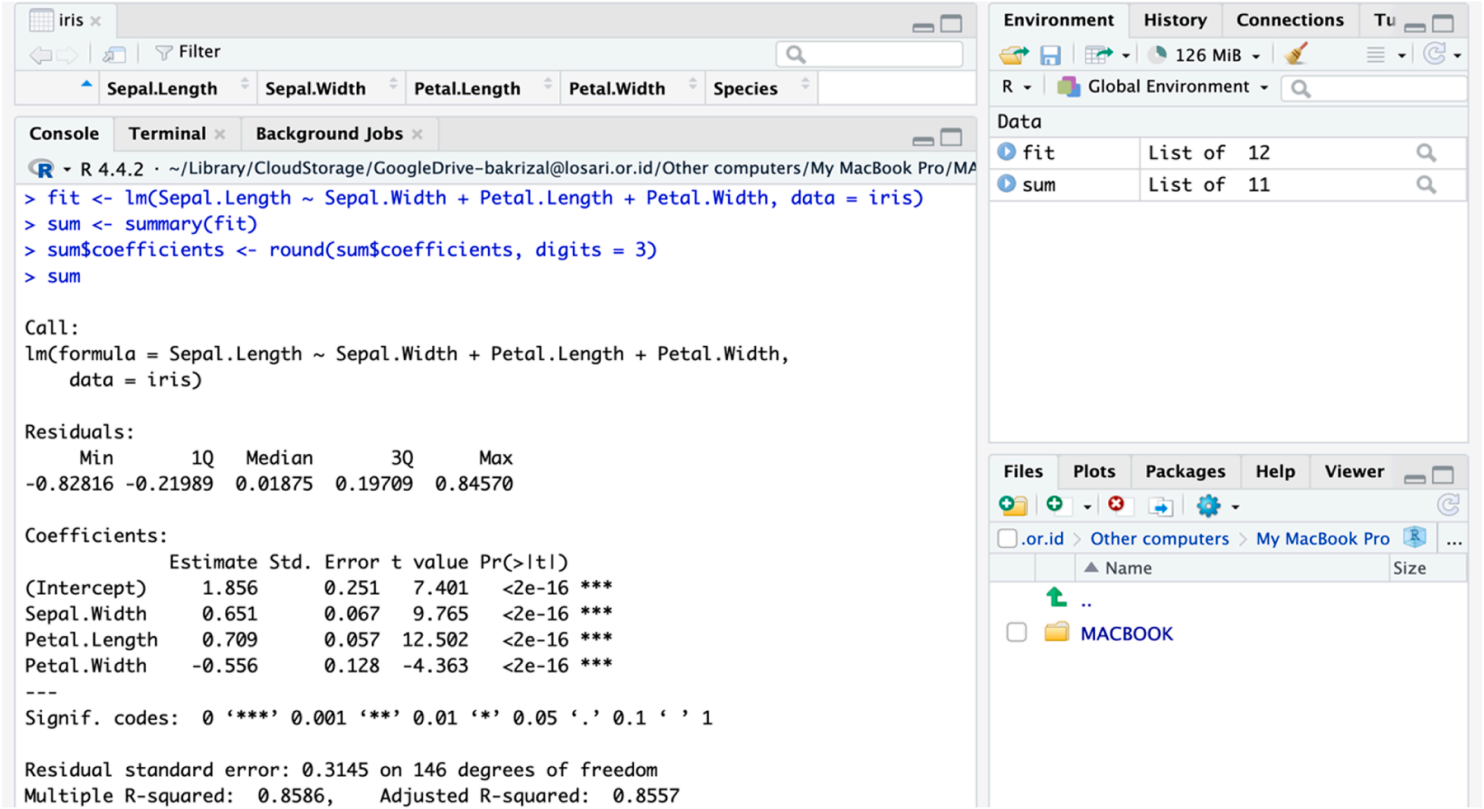
RStudio console output showing regression script and results for the *iris* dataset.

For reproducibility, LOSARI was tested using the same dataset and regression model, running under R version 4.3.1 and Shiny Server version 1.10.0. The analysis can be replicated in any standard R environment with the same versions or accessed directly via LOSARI (https://apps.losari.or.id/lr), which requires no local installation. Detailed procedural steps of the regression analysis using LOSARI are provided in Fig. A.1, Fig. A.2 in Appendix A, and R studio is provided in Fig. B.1 in Appendix B.

A comparison of key outputs is presented in Table 2, confirming that LOSARI produces results identical to R Studio across all tested parameters. This demonstrates that the LOSARI interface does not compromise the accuracy of the underlying R computations while improving accessibility for students unfamiliar with coding.

**Table 2:** Comparison of regression outputs between LOSARI and R Studio.

Parameter / Output	R Studio Result	LOSARI Result	Match Validation
Coefficient ($\beta_{0}$)	1.856	1.856	Yes
Coefficient ($\beta_{1}$)	0.651	0.651	Yes
Coefficient ($\beta_{2}$)	0.709	0.709	Yes
Coefficient ($\beta_{3}$)	−0.556	−0.556	Yes
t-value ($\beta_{0}$)	7.401	7.401	Yes
t-value ($\beta_{1}$)	9.765	9.765	Yes
t-value ($\beta_{2}$)	12.502	12.502	Yes
t-value ($\beta_{2}$)	−4.363	−4.363	Yes

These validation steps ensure that LOSARI’s analytical engine reproduces standard R outputs precisely. By providing both code snippets and dataset references, this study supports transparency, reproducibility, and the FAIR principles (Findable, Accessible, Interoperable, and Reusable) in educational software development.

### Student’s satisfaction using LOSARI in SRL model

As part of the software validation, this study also measured student satisfaction with LOSARI in supporting self-regulated learning (SRL) outside the classroom. Satisfaction was assessed using the End-User Computing Satisfaction (EUCS) model, which consists of four dimensions: content, accuracy, interface, and performance.

Data were collected through an online survey distributed via Google Forms to students who had used LOSARI in statistics courses across four semesters. A total of 777 students participated and provided their perceptions of LOSARI.

**Fig. 6:**
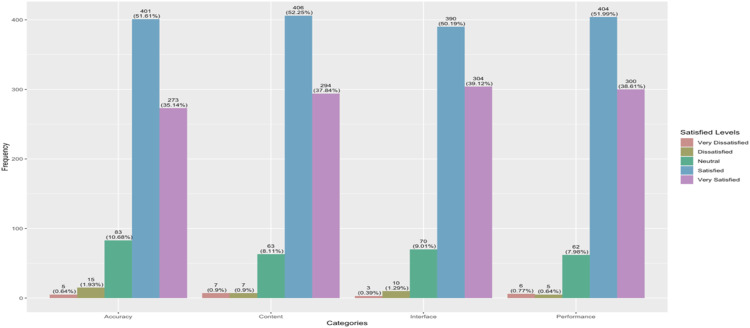
Results of student satisfaction with LOSARI based on the End-User Computing Satisfaction (EUCS) model, showing high satisfaction levels across four dimensions: content, accuracy, interface, and performance.

The results showed high levels of satisfaction across all dimensions. Regarding accuracy, 51.61 % of respondents reported being satisfied and 35.14 % reported being very satisfied with the precision of LOSARI’s statistical outputs. For content, 52.25 % indicated satisfaction and 37.84 % expressed high satisfaction with the relevance, completeness, and usefulness of LOSARI features in meeting user needs. With respect to the interface, 50.19 % of students reported satisfaction and 39.12 % reported high satisfaction concerning ease of use, appearance, and accessibility. Finally, for performance, 51.99 % expressed satisfaction, and 38.61 % reported high satisfaction with LOSARI’s speed, responsiveness, and effectiveness in conducting analyses. These results are summarized in Fig. 6, which presents student satisfaction scores across all EUCS dimensions.

Overall, these findings demonstrate that LOSARI met user expectations in terms of content quality, output accuracy, interface design, and system performance. This aligns with [52], who reported that integrating R-Shiny applications into coursework can enhance students’ comprehension of advanced statistical techniques and increase overall course satisfaction. Similarly, [53] found that students provided positive feedback (e.g., usability, visualization) when learning statistics through interactive simulations developed in R with Shiny. In addition, [54] emphasized that online learning tools and institutional support play an important role in student satisfaction, while [55] concluded that when students embrace technology with enthusiasm and report satisfaction, the technology can be considered effective in promoting student engagement in SRL. Therefore, in general, LOSARI provides strong support for students in SRL, especially in statistics courses.

### Limitations

Despite its innovations, LOSARI still has several limitations that need to be considered. First, the scope of available statistical analyses remains limited to basic methods such as descriptive statistics, correlation, regression, and *t*-tests, although the development of additional statistical methods is still ongoing. Advanced techniques, including design of experiments (ANOVA), structural equation modeling (SEM), machine learning, forecasting, and other methods relevant to business analytics, are not yet supported. Second, LOSARI currently has no direct integration with e-learning platforms, which limits its seamless adoption in formal online or blended learning settings. Third, the video tutorial library is still limited in number and scope, providing guidance only for selected methods.

Fourth, as an online, cloud-based application, LOSARI’s performance depends on both server capacity and network bandwidth. Under heavy traffic or unstable internet connections, users may experience slower response times or temporary disruptions when processing large datasets or streaming video tutorials. Future development will therefore focus on optimizing performance for low-bandwidth environments and strengthening server infrastructure to ensure stable and scalable access for users across diverse learning contexts.

Planned enhancements are directed at addressing these limitations. Future development will expand LOSARI’s capabilities to include advanced methods such as Design of Experiment (ANOVA, MANOVA, etc.), SEM, machine learning, forecasting, and other business analytics tools. Support for multiple languages will also be added to broaden accessibility for global users. Integration with e-learning platforms, including the embedding of theoretical content and comprehensive tutorials, will strengthen LOSARI’s role as a self-regulated learning (SRL) tool. Furthermore, the automatic interpretation feature will be extended with AI-driven algorithms to support more complex analyses. Future studies will also investigate the impact of LOSARI on students’ academic performance under different SRL conditions.

## Ethics statements

All data utilized in this study were collected by researchers adhering to the respective ethical guidelines and without violating privacy rights. No additional ethical approval was required for the use of these datasets in our study

## CRediT author statement

**Rizal Bakri:** Conceptualization, Methodology, Software development, Validation, Formal analysis, Writing-Original Draft, Visualization, Review & Editing. **Eva Boj del Val:** Review & Editing, Supervision. **Basri Bado**: Supervision, Funding acquisition. **Ansari Saleh Ahmar:** Review & Editing, Software validation, Supervision.

## Supplementary material *and/or* additional information [OPTIONAL]

None

## Declaration of competing interest

The authors declare that they have no known competing financial interests or personal relationships that could have appeared to influence the work reported in this paper.

## Figures and Tables

**Table C.1:** Available statistical methods and their corresponding R packages.

Available data analysis methods	R packages used
Basic Statistics	fBasic [35]; GGally [36]; ggplot2 [37]; plotly [38]; nortest [39]; stats [40]; normtest [41]; MVN [42]
Graphs	graphics [40]; plotly [38]
Compare Means	stats [40]; nortest [39]; normtest [41]; ggplot2 [37]; plotly [38]; webr [43]
Correlation	stats [40]; corrplot [44]; gclus [45]; MVN [42]; nlcor [46]; ppcor [47]
Regression	stats [40]; MASS [48]; ggplot2 [37]; Rmisc [49]; nortest [39]; normtest [41]; DescTools [50]; lmtest [51]

**Table D.1:** Examples of LOSARI’s real-time error detection and feedback.

Error Type	Trigger Condition	Notification Message	Suggested Solution
Data Type Error	Non-numeric variable used in numeric analysis	“Selected variable must be numeric.”	Redefine variable type in *Data Preparation → Define Variable*.
Missing Data	NA values detected in selected variables	“Missing data found. Please handle or remove NA values.”	Apply *Filter* or *Imputation* before analysis.
Multicollinearity	High correlation among predictors (VIF > 10)	“Multicollinearity detected.”	Remove or combine correlated variables.
Non-significant Results	p-value > 0.05	Table cell highlighted in red	Review predictor relevance; refine the model.

## Data Availability

Data will be made available on request.

## Appendices

### Appendix A. Steps of regression analysis using LOSARI

This appendix illustrates the procedure of conducting a multiple regression analysis in LOSARI using the *iris* dataset.

Step A.1 Upload the dataset (iris) through the Data Preparation menu. In the Build Model menu, specify Sepal.Length as the dependent variable, and Sepal.Width, Petal.Length, and Petal.Width as independent variables. If advanced settings are required, they can be configured in the More Options section.

Step A.2 Click the Run Analysis button to execute the regression and review the generated output, which includes both statistical results and automatic interpretation.

### Appendix B. Steps of regression analysis using R Studio

This appendix presents the equivalent regression analysis in R Studio for comparison with LOSARI. The console output contains both the R script and the regression summary.
